# A robust method for the detection of small changes in relaxation parameters and free water content in the vicinity of the substantia nigra in Parkinson’s disease patients

**DOI:** 10.1371/journal.pone.0247552

**Published:** 2021-02-24

**Authors:** Krzysztof Dzieciol, Elene Iordanishvili, Zaheer Abbas, Adjmal Nahimi, Michael Winterdahl, N. Jon Shah

**Affiliations:** 1 Institute of Neuroscience and Medicine 4 (INM-4), Forschungszentrum Jülich GmbH, Jülich, Germany; 2 Department of Nuclear Medicine and PET Center, Aarhus University, Aarhus, Denmark; 3 Institute of Neuroscience and Medicine 11 (INM-11), Forschungszentrum Jülich GmbH, Jülich, Germany; 4 Jülich Aachen Research Alliance (JARA-BRAIN)—Translational Medicine, Aachen, Germany; 5 Department of Neurology, RWTH Aachen University, Aachen, Germany; Linköping University, SWEDEN

## Abstract

Alterations in the substantia nigra are strongly associated with Parkinson’s disease. However, due to low contrast and partial volume effects present in typical MRI images, the substantia nigra is not of sufficient size to obtain a reliable segmentation for region-of-interest based analysis. To combat this problem, the approach proposed here offers a method to investigate and reveal changes in quantitative MRI parameters in the vicinity of substantia nigra without any *a priori* delineation. This approach uses an alternative method of statistical, voxel-based analysis of quantitative maps and was tested on 18 patients and 15 healthy controls using a well-established, quantitative free water mapping protocol. It was possible to reveal the topology and the location of pathological changes in the substantia nigra and its vicinity. Moreover, a decrease in free water content, T_1_ and T_2_* in the vicinity of substantia nigra was indicated in the Parkinson’s disease patients compared to the healthy controls. These findings reflect a disruption of grey matter and iron accumulation, which is known to lead to neurodegeneration. Consequently, the proposed method demonstrates an increased sensitivity for the detection of pathological changes—even in small regions—and can facilitate disease monitoring via quantitative MR parameters.

## Introduction

Parkinson’s disease (PD) is a progressive neurodegenerative disorder with rising incidence due to an ageing population [1, 2]. Motor dysfunction in PD is linked to a loss of dopaminergic neurons and the accumulation of iron in the substantia nigra (SN) [2–4]. Consequently, understanding changes in the SN is essential for diagnosis and treatment monitoring. However, this is particularly challenging, due to its small size. Although the underlying mechanisms, i.e. protein misfolding and aggregation, oxidative stress and mitochondrial dysfunction, associated with PD are known and have been studied extensively [5, 6], establishing quantitative biomarkers, crucial indicators for monitoring the disease, has been more difficult. Magnetic resonance imaging (MRI) enables the detection and monitoring of the pathophysiological substrate of the disorder non-invasively, while quantitative MRI gives more sensitive and unbiased information about the tissue properties. Most of the quantitative MRI studies in PD have focused on the evaluation of iron deposition in the SN solely using R_2_* or susceptibility imaging [7–9]. There are very few studies reporting R_1_ changes [10] and, to the best of our knowledge, none investigating total free water content (FW) in this pathology. Recently, Ofori et al. [11] reported an increase in the fractional volume of free water using bi-tensor diffusion. This is, to date, the most important finding related to water content variations in PD patients, since it is also supported by the longitudinal study of Burciu et al. [12] However, this should not be confused with FW since the latter depicts the total MR-visible free water. It is known, that during normal ageing, the concentration of neuromelanin increases in order to facilitate a higher concentration of iron and to protect the cell from its toxicity and ferroptosis [13–15]. In the case of PD, however, as dopaminergic neurons die, iron-saturated neuromelanin is released extracellularly, leading to iron-mediated toxicity, which in turn activates microglia and causes inflammation [16]. Therefore, it is apparent that the multiple pathological substrates of PD require simultaneous investigation of multiple quantitative parameters e.g. T_1_, T_2_* and FW, which–thus far–has not been reported.

Although the aforementioned studies have attempted to investigate the manifestation of the pathophysiology in the SN via quantitative MR parameters, they are based on either manual segmentation or thresholding using contrast provided by one of the employed sequences. This introduces a source of bias, as each sequence is sensitive to different biological changes in the tissue. Therefore, the shape and size of the region-of-interest (ROI) might differ. Furthermore, segmentation of the pathological SN is very difficult, especially in patient cohorts, due to low contrast, insufficient resolution in the MRI images and inevitable partial volume effects caused by the movement of the subject and misregistration. Typically, gradient echo sequences with an isotropic 1 mm in-plane resolution and a slice thickness of 1 or 2 mm are employed to access quantitative water content mapping [17, 18]. However, the SN only consists of approximately 300 voxels–too few for detailed investigation.

It is a common practice to perform manual segmentation of small structures. For example, Keuken et al. [19] used the superposition of voxels selected independently by two researchers. With ultra-high-field MRI providing higher signal-to-noise-ratio and an adequate inter-rater reliability metric (e.g. dice coefficient)—manual segmentation is, in many cases, the most robust approach. Some studies report that the best contrast for revealing the SN is magnetisation transfer contrast (MTC). For example, Bolding et al. [20] compared MTC with T_2_-weighted images using histological brain sections as a reference and concluded that MTC images were more suitable for manual segmentation. This knowledge has been subsequently employed, for instance, by Chen et al. [21] to calculate the volume of SN regions in a healthy cohort. A multi-modal approach, suggested by Visser et al. [22], involved machine learning by means of a Bayesian framework to find the expected appearance of edges on a training data-set. This information was then used to deform an initial rough mesh of the feature of interest, based on intensity profiles across the boundaries. Ali et al. [23] defined the probability density function based on a training data set which was then used (via Bayesian decision theory) to classify the voxels of the target image. In a recent variation of the gold-standard unified segmentation method of Ashburner and Friston [24], where spatially normalised images are partitioned into different tissue probability maps, Lambert et al. [25] utilised a modified multivariate mixture of Gaussians on magnetisation transfer and proton density maps. Recently, Milletari et al. [26] proposed employing convolutional neural networks (CNN) for a fully automated segmentation of the brain. However, although this method is very robust, multi-modal and multi-regional, it does not perform well for small ROIs, including the SN. Finally, Archer et al. [27] managed to train SVM (support vector machine) to detect the pathology in a diffusion-weighted MRI image. The approach demonstrated very good performance; however, a priori delineated data was used. The main aim of this work is to present a method for quantifying the changes seen in the SN of PD patients. The method aims to overcome the dual challenges of low statistics and poor detectability of the area of interest (SN) due to low SNR and insufficient resolution in “standard” MRI techniques. Here, together with contrast enhancement via a joint metric, clustering is used as an alternative to sophisticated correction methods for type-1 errors and to further decrease partial volume effects. As it requires *a priori* knowledge about the existence of the pathology and does not provide an accurate contour, it cannot be treated as a typical segmentation method. It is noted that, very often, it is not segmentation of the SN or its topological contours that are important but rather statistically significant deviant voxels; the method described herein delivers such voxels. Due to its simplicity, the described methodology can offer an interesting alternative to existing strategies. Moreover, it is, to the authors’ knowledge, the first study investigating all three quantitative parameters i.e. T_1_, T_2_* and FW together in a PD cohort.

## Material and methods

### Study cohort

A total of 33 participants, 18 with PD and 15 age- and gender-matched healthy controls were scanned in this study. The PD patients were recruited in collaboration with the Department of Neurology, Aarhus University Hospital, Denmark, and fulfilled the U.K. Parkinson Disease Society Brain Bank criteria for PD [28]. The healthy control subjects had no known neurological or psychiatric disorders or any MR visible manifestation of other pathological conditions. Note that, the information regarding the full medical history of patients was not available to the authors, and, therefore, this study is not intended to give insight to quantitative parameters response to treatment. The demographic data of the cohort are shown in Table 1. After receiving detailed study information, written consent was acquired from each subject or their legal representative. The study was approved by the research ethics committee of Region Midtjylland and The Danish Data Protection Agency and was conducted in agreement with the Declaration of Helsinki.

**Table 1:** Demographic and clinical characteristics of the study cohort.

*Demographic and clinical variables*	*Healthy Controls*	*Patients*
Age (median, min, max, std)	66, 52, 76, 8.5	68, 53, 81, 8.5
Gender Male: Female	8:7	11:7
Education level in years (median, min, max, std)	15, 9.5, 23, 3.2	14, 6, 19, 3.5
PD duration in years (median, min, max, std)		9, 7, 15, 3
Hoehn & Yahr staging (number of patients)		2 (8), 3 (6), NA (4)

### Measurement protocol

Participants were scanned with a Siemens multi-slice, multi-echo RF spoiled gradient-recalled echo sequence (GRE) optimised for the quantitative mapping of cerebral water content [17, 18]. For details relating to the applied water mapping protocol, the reader is referred to Neeb et al. [17], Gras et al. [29], and Abbas et al. [18] but a general overview will be given here. Unless stated, all sequences used were standard.

As a foundation for further calculations, a proton-density-weighted image (Siemens GRE sequence) was acquired using partially parallel acquisition (GRAPPA) with an acceleration factor of 2 and 24 auto-calibration lines, TR = 1800 ms, TE = 5.8 ms, FA = 40° and bandwidth = 210 Hz/pixel. Two interleaved concatenations (32 slices each) were used, resulting in a gap-free acquisition. This finally led to 64 transverse slices of 2 mm thickness and 1 mm in-plane resolution, measured in 6 minutes.

The transmit profile (B_1_^+^) was measured via a multiple flip angle technique (MFA) [29–31]. This technique employs 4 echo-planar images (EPI) with different FAs (30°, 60°, 90° and 120°) with a TR of 20 seconds (for full relaxation), acquired in 1.5 minutes.

For the estimation of T_1_ relaxation times, the two-point method was used [18, 32] employing two acquisitions, i.e. proton-density-weighted GRE and T_1_-weighted GRE (TR = 500 ms, TE = 5.8 ms, FA = 90°) along with B_1_^+^ information. The total time required for the two-point method acquisitions was 2 minutes.

Receiver profile correction (B_1_^-^) was achieved using a low-resolution GRE sequence. This was performed through two measurements, first using the phase array and then using the body coil for the signal reception. The same parameters were used in both cases, i.e. TR = 500 ms, TE = 5.8 ms, FA = 40° [30] adding 1 minute to the total acquisition time.

In order to sample the T_2_* decay, a 3D GRE was acquired (3 minutes) with 8 echoes [18] (1x1x2 mm, TE_1_ = 2.3 ms, ΔTE = 2.3 ms). This also allowed the T_2_* decay of the proton-density-weighted scan to be corrected.

For all sequences, the same orientation and field-of-view were used. All mentioned parameters were as presented in Abbas et al. [18].

Additionally, a T_1_-weighted 3D, magnetisation preparation rapid gradient-echo sequence (MPRAGE) was acquired and used for further registration to a standard MNI template (Montreal Neurological Institute [33]). This scan was acquired with the following parameters: TR = 2250 ms, TE = 3.37 ms, TI = 1100 ms, FA = 15°, FOV = 256x256x256 mm, with isotropic resolution of 1 mm and 176 sagittal slices. The T_1_-weighted 3D sequence added 8 minutes to total acquisition time. In the end, the whole acquisition protocol took 18.5 minutes for each subject.

### Estimation of relaxation parameters and total free water content

Following the acquisition of the MR images, FW, T_1_ and T_2_* were estimated and additional corrections, as presented by Abbas et al. [18], were applied. However, after the corrections for T_1_, T_2_* and transmit/receiver profiles, the proton-density-weighted scan is not yet ready to be treated as a quantitative water content map and requires further correction for receiver bias profile [18]. This was achieved using the well-known linear relation between proton density contrast and T_1_ relaxation time in certain brain regions (60 ms > T_2_* > 50 ms).

Finally, instead of choosing arbitrary voxels within the cerebrospinal fluid (CSF), a more sophisticated procedure for normalisation was used [18, 34]. Valid regions were located based on T_1_, T_2_* thresholds and stability in terms of their transmit profile. The normalisation factor was then computed via the weighted average across the stable regions.

### Registration

**Fig 1:**
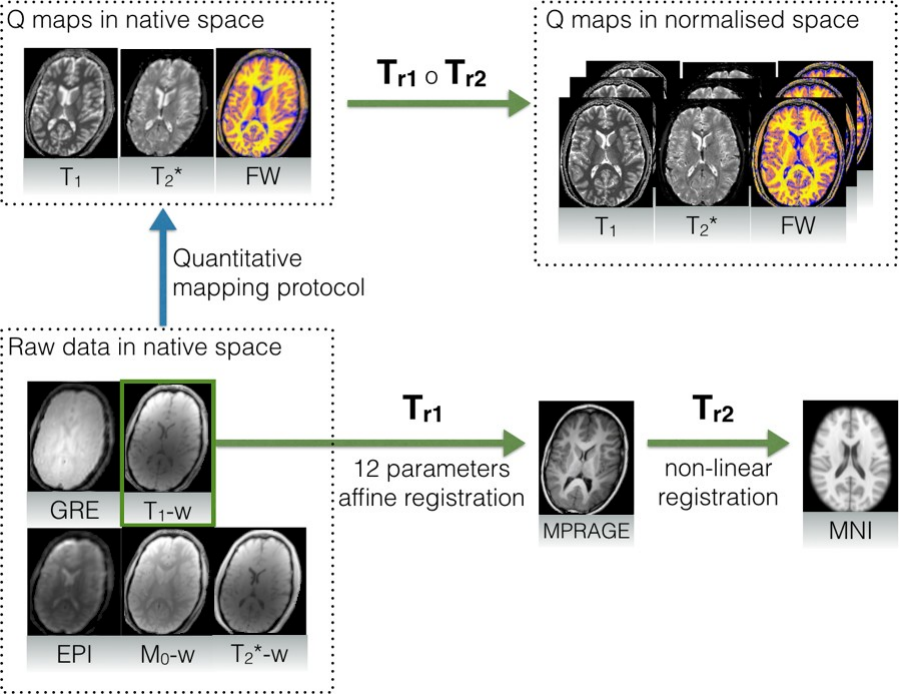
Registration procedure for quantitative maps with the assistance of an MPRAGE image for nonlinear alignment. Raw data is registered to the additional MPRAGE image (T_r1_) which is then non-linearly deformed (T_r2_) to match the MNI template. Composition of both deformations provides the final transformation.

Quantitative maps were registered to a common template (MNI152 1x1x1 mm) by means of the combination of linear and non-linear registration methods. The pre-processing chain is presented in Fig 1.

Twelve-parameter affine linear registration was applied to match the quantitative maps with the MPRAGE image. In order to stay in the same modality, T_1_-weighted acquisition was considered as being most similar to MPRAGE. Good inter-subject matching was obtained by non-linear registration of the MPRAGE image to the MNI template. Here, diffeomorphic demons registration was applied. This step was performed using an in-house solution based on the method presented by Vercauteren et al. [35], with all the computationally exhaustive elements such as gradient calculations, convolution and interpolation implemented on a GPGPU (OpenCL).

**Fig 2:**
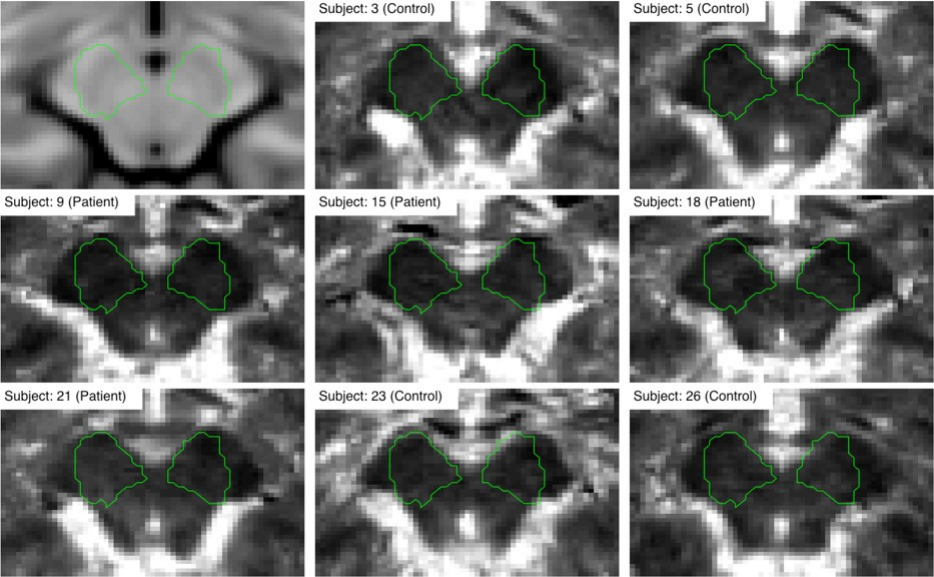
Registration performance for the example T_1_ quantitative image. The green outline represents the rough contour of the search region.

Finally, by defining T_r1_ as an intra-subject, linear transformation between MPRAGE and quantitative maps, and T_r2_ as a non-linear transformation between MPRAGE and the MNI template, the complete transformation was constructed as a composition of both: *T_r_* = *T*_*r*1_ ∘ *T*_*r*2_. A single registered slice, together with the reference, is shown in Fig 2.

### Region of interest definition

Although the exact location and shape of the SN was not assumed *a priori*, a rough contour (ROI) was defined in order to highlight the changes around its expected location. To make analysis easily replicable, a radiologist manually delineated the SN region on an MNI template using the ITK-SNAP toolbox [36]. The ROI was then dilated with a spherical kernel to contain around 1000 voxels. The final contour is presented in Fig 2. Note, that the contour can be in fact arbitrary since it acts only as additional mask revealing area of interest around the SN.

### Statistical analysis

Following registration, global statistical analysis was performed by calculating the average values of the quantitative parameters of white matter (WM) and grey matter (GM) in the whole brain and inside the ROI. Voxels belonging to WM and GM were selected based on SPM templates [24], since all the volumes were already registered to a common coordinate space. Partial volume effects were suppressed by using a 0.95 threshold for the SPM template. Note that, this method does not specifically consider possible misregistration, and consequently, the voxels close to the borders may still be affected by partial volume, leading to a bigger variance of the data used for statistical tests. This is a significant issue, particularly in terms of the correction of type-1 errors. Every method that corrects for family-wise-errors is based on certain assumptions. For example, in the case of Random Field Theory, there is a dependence between Euler Characteristics and data topology i.e. smoothness, number of resells, shape and size of the volume [24]. Hence, in the case of a small signal-to-noise ratio, an adjustment of the Z-score threshold is required, which ultimately reduces confidence in the results. In the following section, an alternative approach, which is based on analysis of raw p-values distributions, will be described.

First, the quantitative parameters were abstracted into a metric estimator, *E*, which was defined as either the value of T_1_, T_2_*, FW or combination of all three:

$$E^{joint}=FW/n_{FW}+T_{2}^{*}/n_{T_{2}^{*}}+T_{1}/n_{T_{1}}$$ (1)

$$E^{T_{1}}=T_{1}$$

$$E^{T_{2}^{*}}=T_{2^{*}}$$

$$E^{FW}=FW$$

where normalisation factors (*n*) are defined as CSF values for the corresponding modalities, taken from calculated quantitative maps. Note that, this way, the weighting is strongest for FW, while T_1_ with T_2_* are acting as contrast-enhancing factors, as long as they follow the same trend. Therefore, firstly, the standard metrics, i.e. $E^{T_{1}},\;E^{T_{2}^{*}}$ and *E^FW^* must be checked and then applied to the joint metric with appropriate signs enhancing the final contrast.

For each metric, two 4D volumes were created—a first one, where each voxel in space was characterised by a vector of 15 values from co-registered controls (*E*_*c*_) and a second, where each voxel was characterised by a vector of 18 values from co-registered patients (*E*_*p*_), as presented in. In order to reduce the possible effect of misregistration, voxels with a signal variation that was too high were eliminated. The exclusion criterion was based on the standard deviation. If a vector from either a control or a patient had at least one value deviating from the mean by more than four standard deviations, then both vectors, and hence the voxel itself, were eliminated from the analysis. This reduced the number of voxels for analysis by a factor of 2 (from around 1x10^6^ to 0.4x10^6^). The final mask can be defined simply as a logical conjunction of a WM mask, GM mask and voxel elimination mask.

A voxel-wise comparison was performed using a simple two-means, one-tailed t-test with a null hypothesis stating "metric has not decreased". The p-value threshold for the null hypothesis was set to 0.05 and the alternative hypothesis H_1_ binary map was calculated. There, high values represent the regions rejecting the null hypothesis. This map was further processed in order to eliminate small clusters using an arbitrary threshold of 27 voxels, which corresponds to the 3x3x3 cube, normally considered as noise limit for segmented data [37]. Here, an additional step was performed in order to further reduce the influence of misregistration and type-1 errors at the same time. With the assumption that the highest misalignment takes place at the interfaces between GM, WM and CSF, the H_1_ map was labelled with 18 connected components (ignoring weak connections at voxels corners). For each cluster, following Oriani et al. [38], solidity was defined as a ratio between the convex hull volume and the total volume. Clusters with very low solidity (0.1) were subsequently removed from the analysis. Finally, all clusters were sorted from biggest to smallest.

Additionally, the differences between estimators were defined as in Eq (2):

$$d\left(E\right)=\bar{E_{p}}-\bar{E_{c}}$$ (2)

Where $\bar{E_{p}}$ and $\bar{E_{c}}$ are the average values of estimators in patient and control vector respectively.

The histograms of *d(E)* were computed both globally and for the selected ROI. This was useful for the observation of signal variation around the SN since hypothesis testing only provides a binary map and p-value distribution.

## Results

**Fig 3:**
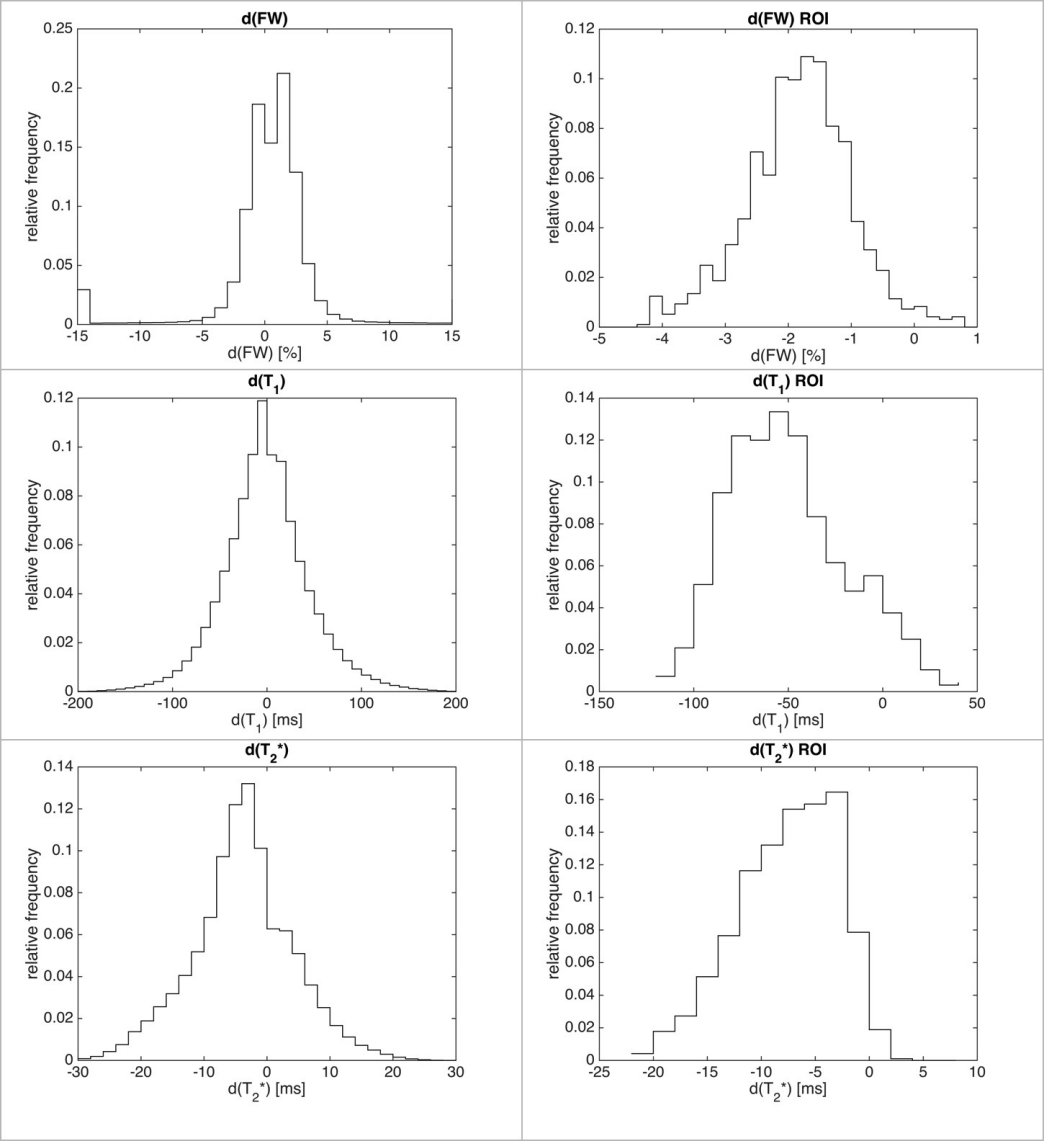
Normalised histograms of differences for all voxels (left column) and inside the ROI (right column). The shift towards the negative values is more apparent inside the ROI. Notice that histograms are cropped for readability and hence do not show outliers.

Normalised histograms of the differences between the estimators for each quantitative parameter globally and within the ROI are presented in Fig 3.

Globally, the histograms were either symmetric (FW, T_1_) or eventually slightly shifted towards the decrease of the metric (T_2_*), indicating a very small value of average change for a given metric. Any attempt of quantification based on the global average failed (Table 2).

**Table 2:** Mean values for water content and relaxation parameters for all GM/WM voxels and ROI.

Quantitative variables	GM				WM			
	Global		ROI		Global		ROI	
	Controls	Patients	Controls	Patients	Controls	Patients	Controls	Patients
FW [%]	83±1	83±1	81±1	79±2	73±2	73±2	77±1	75±2
T_1_ [ms]	1501±40	1469±55	1422±77	1375±91	1169±85	1171±86	1167±73	1172±79
T_2_* [ms]	56±5	54±7	36±3	36±6	48±5	48±5	40±4	41±6

Even when only considering the region in the vicinity of the SN (ROI), very little change was found and any change was practically hidden within the range of standard deviation. Although the histograms are shifted towards negative values inside the ROI, the non-gaussian shape suggests that misregistered voxels play an important role in this small region.

**Fig 4:**
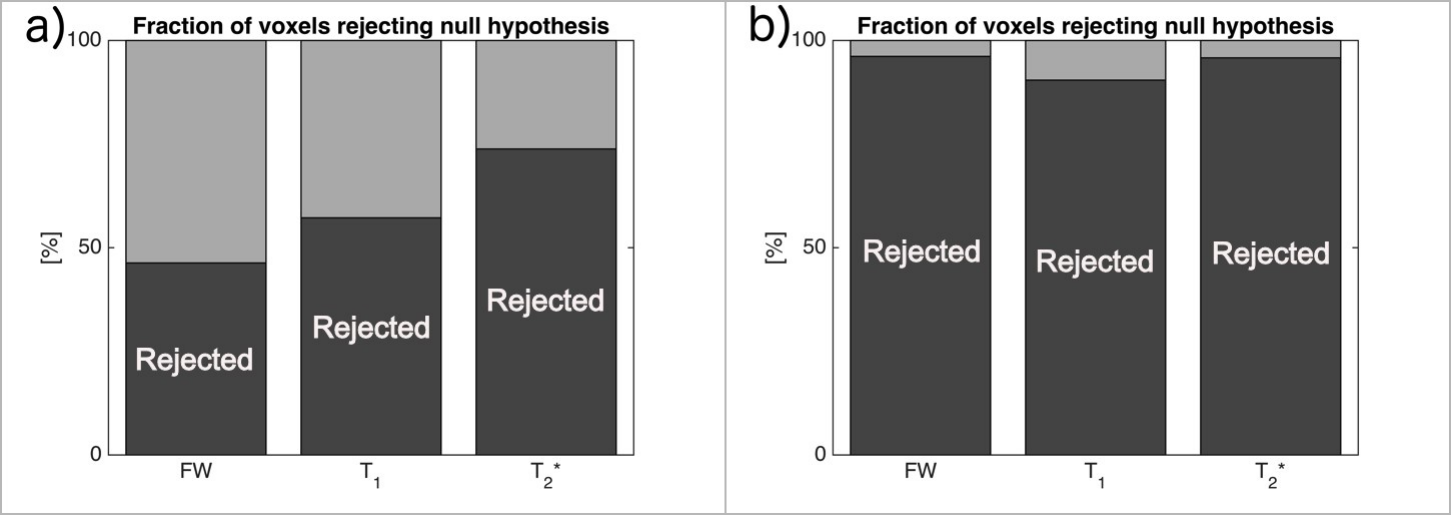
Fraction of voxels rejecting the null hypothesis (dark-grey) with a p-value of 0.05, hence supporting the statement that “the metric has decreased” for all voxels (a) and inside the ROI (b).

Voxel-based statistical analysis showed a similar result, as long as all voxels were considered. In Fig 4, the dark-grey shaded area represents the fraction of voxels rejecting the null hypothesis, therefore confirming the decrease in the metric with a p-value at 0.05. In fact, the commonly used value of 0.05 is far too permissive in the case of multiple comparisons, and therefore, around half of the voxels in the one-tailed t-test reject the null hypothesis. This is clearly visible in Fig 4A for each metric, except the T_2_*, which shows different behaviour, supporting, in this case, the statement that T_2_* values decreased globally. Inside the ROI, this statement is supported even more strongly and for each metric. Binary histograms, representing the fraction of voxels rejecting the null hypothesis, as shown in Fig 4B, indicate that most of the voxels decreased in value, even if the difference was small. Although the magnitude of change is not provided with enough significance, it is clear that the values of T_1_, T_2_* and FW, are reduced for most of the voxels inside the ROI.

**Fig 5:**
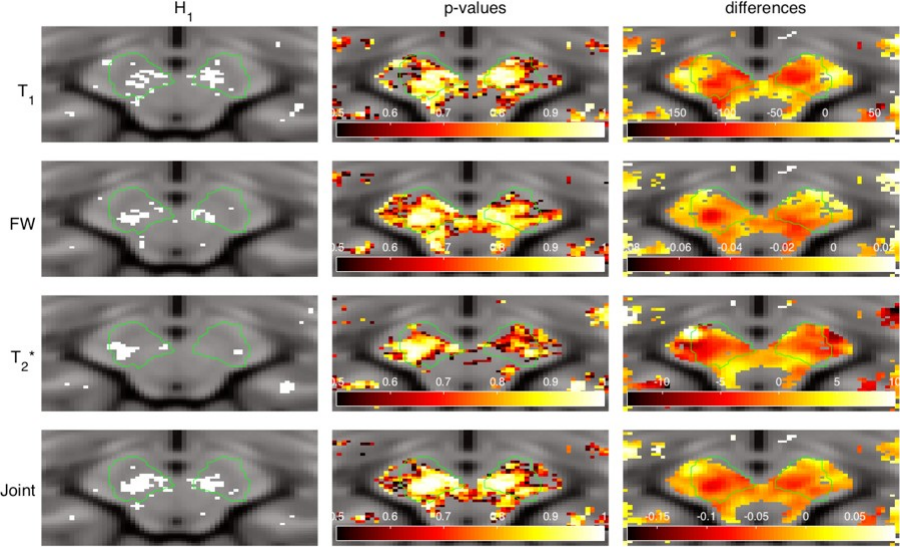
Null hypothesis rejection indicator (first column, white = rejected), raw p-values (middle column) and differences between estimators (last column) for each metric selected for the analysis.

The H_1_ binary map shown in Fig 5, together with the corresponding distribution of differences and raw p-values for each metric, reveal the bilateral clusters of voxels rejecting the null hypothesis in the vicinity of the SN. The distribution of differences (last column) shows the greatest contrast in the case of the joint estimator. Moreover, the detected clusters are bigger and more homogenous than those revealed by individual metrics.

**Fig 6:**
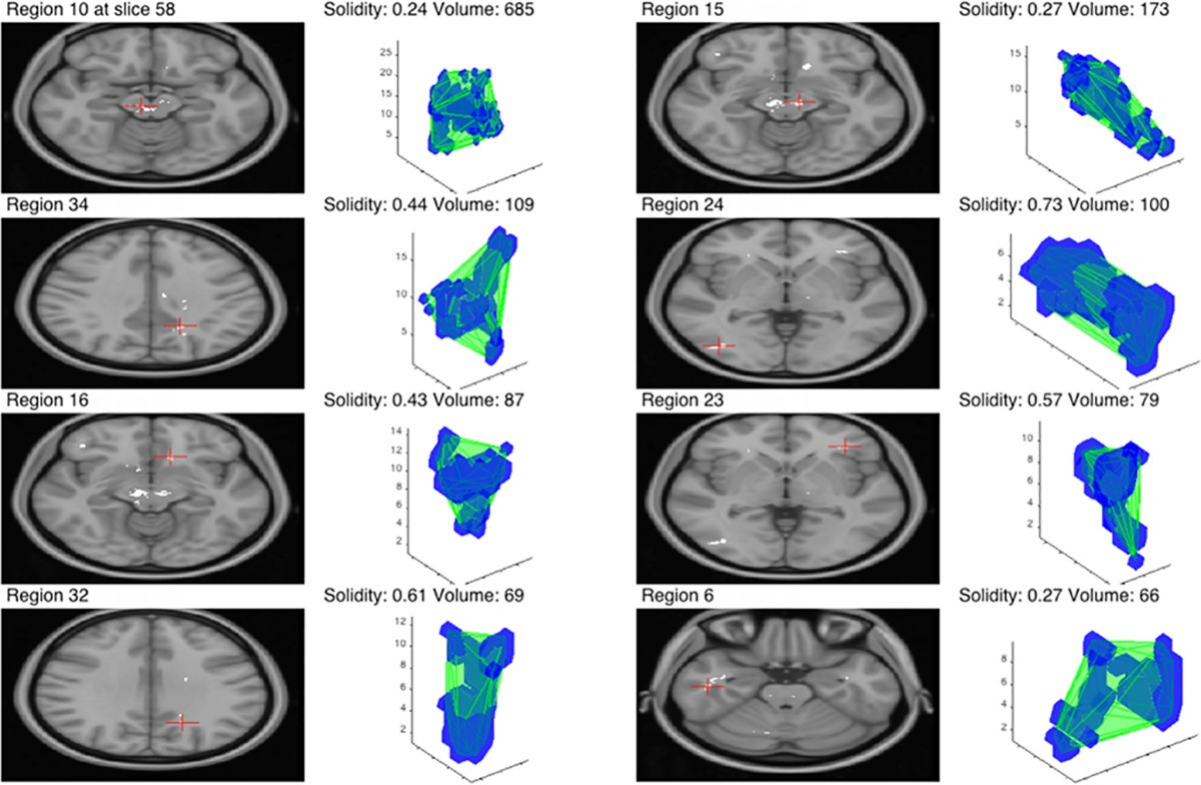
Eight biggest clusters for the joint metric. The green outline in 3D visualisation represents the convex hull of the cluster and the corresponding central slice is shown on the left with a red cross indicating the centre of the mass.

Following the removal of any small or irregular clusters, based on solidity, it can be seen that the biggest clusters are the two located in the vicinity of the expected location of the SN. This is visualised in Fig 6. Notice a very small number of remaining clusters and a significant difference in the volume between the first two clusters and the rest. The same situation was observed for each metric with the exception of T_2_*, where the decreased value was distributed across large regions within the whole brain and more clusters were detected globally. A summary of this can be seen in Table 3. The consistent location of the two biggest regions inside the expected location of the SN for T_1_, FW and, most importantly, the joint estimator, confirms that the observed decrease in parameters is not the result of random effects.

**Table 3:** Summary of detected clusters of voxels in the whole brain.

Metric	Number of clusters	Number of clusters after elimination	Were clusters within SN search region biggest?
T1	99	42	Yes
T2*	129	51	No
FW	43	18	Yes
Joint	40	17	Yes

**Fig 7:**
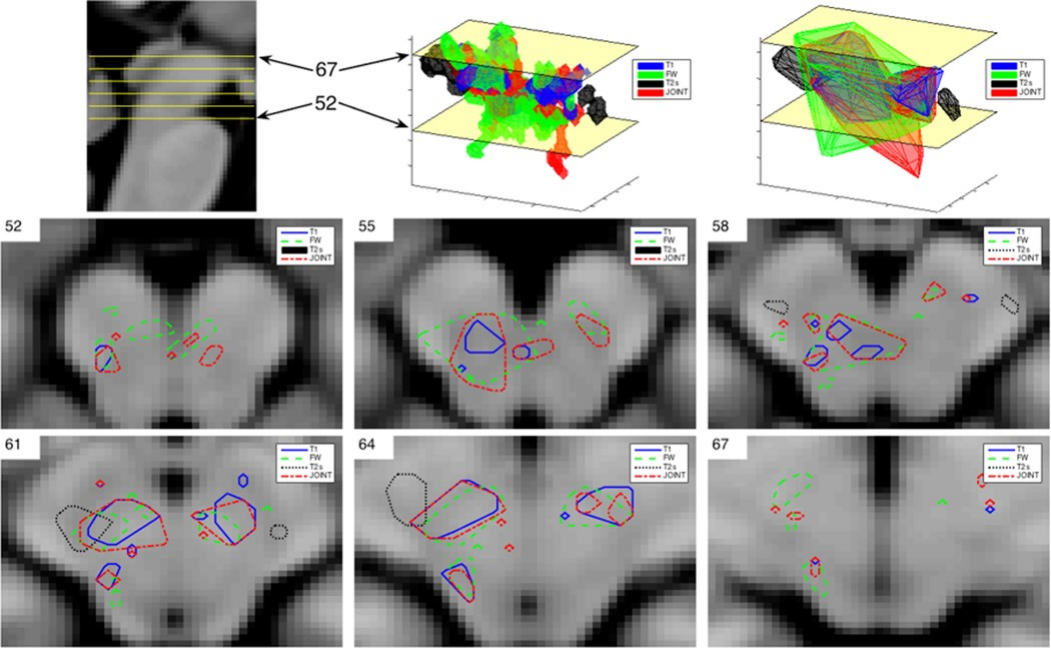
H_1_ clusters overlap for the SN region. In the top row, sagittal view (first picture) is presented for orientation, together with 3D visualisation of SN clusters (middle) and their convex hulls (last picture). In the second and third row, outlines of SN regions detected by each metric are shown for selected slices.

There is a good overlap (around 80%) between the clusters detected by each metric within the SN search region. In *Fig 7*, for clarity, the overlap between the convex hulls and 2D outlines are shown. The clusters, according to Duvernoy’s Atlas of the Human Brain Stem and Cerebellum *[39]*, span across several brain regions located in the midbrain, i.e. the ventral tegmental area (VTA), cerebral peduncle (CP), retrorubal field (A8), and the anterior and posterior SN (see Table *4* for details). Notice that, while T_2_* clusters are smallest; they are located precisely inside the SN. Others, including the joint estimator, are extending outside the SN and towards the centre of the midbrain.

**Table 4:** Regions of the brain overlapping with H_1_ clusters.

Metric	Right hemisphere	Left hemisphere
T1	SN post.; CP	SN post.
T2*	SN ant.; VTA; CP; A8	SN ant.; VTA
FW	SN ant.; CP; VTA; A8	SN ant.; VTA
Joint	SN; VTA; CP; A8	SN; VTA

## Discussion

### Segmentation and statistical analysis

Assuming that the region of the SN is affected by PD and that the pathological changes are reflected in the quantitative MR parameters, the voxel-wise statistical approach presented here was able to identify the pathological regions. The voxels rejecting the null hypothesis, hence indicating a reduction in the metrics, are contained within the ROI. This allows conclusions to be drawn about the decreased MR parameters i.e. T_1_, T_2_* and FW in the vicinity of the SN, i.e. VTA, CP, A8 and the SN itself. Although the approach relies on the assumption of the existence of the pathology, this disadvantage is balanced by the simplicity of the method itself and the good final contrast. This can be seen in Fig 5, where the H_1_ clusters are located inside and in the vicinity of the SN. More advanced strategies, i.e. based on machine learning [22] or employing additional modalities [40], may provide the valid contour, but this comes with the cost of higher implementation complexity and reduced robustness. On the other hand, a manual approach, which can be accurate, especially with a higher SNR provided by ultra-high-field MRI, is still prone to human error and is extremely time inefficient [19]. The proposed method uses no spatial priors and initially checks the number of voxels with changed signal intensities. Therefore, it counteracts the low statistical power and the problem of signal averaging or partial volume effects inside the ROI. Moreover, it does not require a sophisticated approach for multi comparison correction–this is bypassed by clustering and voxel elimination step–and hence, a standard p-value threshold can be used, even if it initially results in a large number of sparse voxels rejecting the null hypothesis.

Although this method may not provide the exact contour of the SN, it indicates the direction of change for a given metric. It is shown that, although variations in quantitative parameters were small, and hence not detected by global statistics, voxel-wise analysis reveals a cluster consistent with decreased MR parameters. This cluster coincides with the pathological region of the SN. In future studies, the size of the cluster could potentially provide a better marker for monitoring disease progression than the average value of a given quantitative parameter, which can easily be disturbed by partial volume effects. Clearly, the existence of multiple clusters outside the ROI, as seen in Fig 7, might be disturbing, and this is the biggest disadvantage of the method. Without *a priori* knowledge of the pathomorphology of the disease, it is impossible to draw conclusions. In the case of the presented work, the authors could not interpret the smallest clusters distributed sparsely among the brain. However, two clusters located in the vicinity of the SN were much bigger, i.e. ~700 voxels vs ~100 voxels, hence giving confidence in the results.

### Relaxation times

The shortening of T_1_ and T_2_* times in the SN coincides with most of the findings in the literature [9, 10, 41] and can be explained based on the pathophysiological substrate underlying the disease. Dopaminergic neuronal loss in the SN pars compacta causes T_1_ shortening [5], while iron and Lewy body accumulation results in both lower T_2_* and T_1_ values [42]. However, there are some studies that contradict these findings. For example, Reimão et al. [43], found no significant difference in T_2_* between controls and patients at baseline and after 2–5 years of disease duration. However, there is evidence that iron accumulation happens years before the symptomatic manifestation of the disorder and by the time clinical symptoms become apparent, half of the neurons are already dead [43, 44]. Nevertheless, attention is drawn to the small sample size and manual segmentation as limitations of this study. Similarly, Menke et al. [40] were not able to find any significant changes in T_1_ values using DESPOT1. Their sample size consisted of only 10 patients and 10 control subjects. Hence, the low statistical power might have been a hindrance for detecting the manifested changes.

In the presented work, it is shown that T_1_ decrease extends outside of the area of the SN and mostly covers the ventral tegmental area, as well as the retrorubral field and cerebral peduncle. Similarly, Baudrexel et al. [10] have shown T_1_ changes beyond the SN in the midbrain regions of PD patients. Interestingly, VTA and the retrorubral field contain dopaminergic neurons and have been shown to degenerate in PD [45].

### Total free water content

Although numerous studies have explored the behaviour of water diffusion in the SN of PD patients using DTI, this is, to the best of the authors’ knowledge, the first attempt to investigate total water content in this region using relaxometry. Many authors have reported decreased fractional anisotropy in the SN, reflecting neurodegeneration [46, 47]. Recently Ofori et al. [11], demonstrated a longitudinal increase in free-water using a bi-tensor diffusion model. At first glance, these data contradict the finding of lower total water content values presented in this work, however, the difference between the physical phenomena observed with each technique must be considered. Ofori used two terms in the model equation: C_tissue_ and C_water_—modelling grey matter or a single bundle of white matter and a fractional volume of free water, respectively. Therefore, C_water_ is mainly influenced by the extracellular water molecules, while the water content quantified in the present work depicts total free water content both in the extra- and intracellular compartments. Later, Guttuso et al. [48], replicated the results of Ofori’s study, but with longer disease duration, and showed the increase of fractional free water only takes place in the anterior part of the SN.

The total free water content measured in the presented study is a sensitive measure of oedema. At a glance, one might think that increased extracellular fluid should also be reflected in the increased FW. However, considering the fact that water is the major source of the MR signal in the tissues, and grey matter has a higher FW content than white matter [49, 50], neuronal cell loss should lead to a reduction in total water. Therefore, it is possible that the increase in extracellular water might not be enough to compensate for the decrease in total water content caused by the disruption of the deep grey matter integrity.

### Limitations and future work

As previously stated, the assumption of the existence of pathology at a severity sufficient to be manifested by a change in quantitative parameters is the main requirement of the proposed method. Furthermore, the contour of the region does not necessarily represent anatomical boundaries, but rather the area in which pathological changes occur, which could be either bigger or smaller than the actual anatomically-defined structure. The small sample size is another limitation of this study. However, the proposed approach uses the affected voxels to evaluate the changes and is actually more robust for the low statistical power encountered by having a relatively small number of subjects. Nonetheless, in the future, it would be desirable to introduce a larger cohort, where the mean values of the quantitative MR parameters are also significantly changed, making it possible to cross-validate the presented voxel-wise statistical approach. Note that, to date, the method has only been tested on a quantitative dataset acquired with a specialised water mapping protocol, and extra care was taken for proper normalisation and correction against inhomogeneities (as in [18, 29]), therefore, the cohort size is small and cannot be easily extended by using publicly available PD data. In the future, this proof-of-concept should be generalised and applied to multiple, large datasets, including different pathological states. In this case, any quantitative metric could be used, with the important condition, however, that pathology actually exists. Otherwise, since the initial threshold for p-values distribution and filtering the segmented H_1_ image (see method section) are rather permissive, it might lead to the incorrect interpretation of results. Moreover, the combined metric will not always be beneficial or even possible to compute at all, especially when there is no (or only one) pathophysiological connection between the parameters. However, in specific cases, like the one presented in this work, a combination of weighted parameters highlights the importance of detected clusters, as presented in Table 3. It would be interesting to compare the existing segmentation methods directly with the contour given e.g. by a H_1_ binary map (Fig 5B). Note however, that in many cases, especially when the segmentation is trivial and changes are apparent, it will be probably easier to segment manually or rely on simple thresholding. On the other hand, even if delineation is easy, statistical analysis might provide only weak; or no proof for (H_1_) hypothesis. In such a case, the presented method would be still advantageous, since the data clustering is taken into account.

Therefore, a longitudinal study including disease progression and analysis of the evolution of the affected region is the ultimate goal for forthcoming work.

## Conclusions

In this study, a new approach is introduced which was able to demonstrate the decrease in all measured quantitative parameters; T_1_, T_2_* and FW, in the clusters of voxels reflecting the shape and the location of the pathological region. This was achieved without the *a priori* spatial constraint of a manually segmented mask. The proposed method overcomes the limited spatial resolution, small number of subjects and poor statistics "per subject" (few voxels of interest). It is expected that this method will help to provide a better understanding of the underlying mechanisms of PD via the measurement of quantitative MR parameters. The observed decrease in relaxation parameters is in agreement with the literature and it is speculated that the reduced FW is most likely due to neuronal cell death. This insight gives an indication of changes both in intra and extracellular space and supplements previous findings showing increased fractional water content in the SN of PD patients.

Finally, the presented approach can be abstracted to a more general method–that of "if-instead-of-how-much" and, by selecting an appropriate null hypothesis, easily extrapolated to other pathologies affecting different brain regions, even if completely different metrics are used as imaging markers.
